# Radionuclide molecular imaging of abdominal aortic aneurysms for risk stratification and non‐invasive therapy assessment

**DOI:** 10.1002/ctm2.386

**Published:** 2021-05-01

**Authors:** Richa Gandhi, Marc A. Bailey, Charalampos Tsoumpas

**Affiliations:** ^1^ Leeds Institute of Cardiovascular and Metabolic Medicine School of Medicine University of Leeds Leeds UK

Dear Editor,

In this letter, we explore the potential role of radionuclide‐based molecular imaging modalities, such as positron emission tomography (PET) and single‐photon emission computed tomography (SPECT), in evaluating abdominal aortic aneurysms (AAA). We further discuss how the developments of promising radiotracers in combination with ultra‐sensitive PET and SPECT scanners may enable the assessment of AAA formation and progression and consequently patient risk stratification and therapeutic interventions.

AAA is an asymptomatic, irreversible, focal enlargement of the abdominal aorta proceeding to rupture and eventually resulting in patient death. Patients with early‐stage AAA do not exhibit evident signs or symptoms; hence, AAA is typically diagnosed during incidental health assessments or through dedicated screening programmes, such as those implemented in the USA, the UK and Sweden. Although many studies are conducted to identify more reliable and informative risk factors of AAA rupture, aortic size is the only marker utilised in clinical practice, with elective repair offered to prevent rupture once an AAA enlarges to the intervention threshold of 5.5 cm. The aortic diameter is a straightforward and convenient parameter to extract using ultrasound scanning (USS) but may not consistently correlate with prognosis, as large AAA may remain intact, while small AAA may rupture.^1^

Much of our knowledge of human AAA pathology stems from aortic tissue obtained during surgery (i.e., late‐stage disease); therefore, theories based on findings corresponding to these tissues may not accurately reflect early‐stage disease pathology, when an effective pharmacological treatment may be offered. Indeed, one of the most significant barriers to overcoming late‐stage detection of AAA is the ability to detect early‐stage molecular mechanisms that precede physical disease manifestation. Patient management can be complicated to navigate based solely on the risk of AAA rupture as predicted using USS for aortic diameter measurements; in this case, molecular information yielded by PET and SPECT may improve the management and treatment approaches for AAA by providing more comprehensive information on the molecular processes occurring within the aortic wall that drive an aneurysm towards growth and rupture as opposed to stability. These molecular‐level data make PET and SPECT imaging attractive tools for the purposes of early risk stratification and the assessment of responses to effective low‐risk non‐invasive therapeutic interventions as adjuncts to USS‐based screening and surveillance.

Basic research in AAA pathobiology continues to generate important preclinical data and novel imaging approaches to support clinical advances. Undoubtedly, the establishment of reliable and unequivocal radiotracers remains the ultimate objective of any radionuclide‐based molecular imaging study. The widely used PET radiotracer [^18^F]fluorodeoxyglucose ([^18^F]FDG) has been shown to correlate with inflammatory markers in AAA (Figure 1); however, its uptake exhibits inconsistent correlations with pathological and clinical features of AAA.^2^, ^3^, ^4^ Thus, alternative radiotracers may be more useful as indicators of molecular events, with implications for more precise AAA risk stratification and personalised interventions. For example, [^18^F]‐ sodium fluoride ([^18^F]NaF) uptake is increased prior to pronounced aortic expansion in the angiotensin II‐induced AAA murine model, reflecting microcalcification, which may indicate an alternative biological process that can offer a surrogate signal of an effective therapy.^5^ Moreover, [^18^F]NaF localises in regions of microcalcification in patients with asymptomatic AAA, acting as a susceptibility marker of aneurysm growth and rupture and yielding proof‐of‐concept data for the practicability of a PET radiotracer other than [^18^F]FDG for risk stratification in patients with AAA (Figure 2).^6^ Meanwhile, in the angiotensin II‐induced AAA mouse model, [^18^F]fluorothymidine demonstrates greater uptake in early‐stage AAA than in late‐stage AAA, reflecting an early period of proliferative activity in AAA formation and potentially growth, opening up the potential for anti‐proliferative therapies that may be administered early in the disease course (Figure 3).^7^ In the porcine pancreatic elastase‐induced AAA murine model, early‐stage [^64^Cu]DOTA‐ECL1i uptake, which reflects binding to chemokine receptor 2, may facilitate rupture prediction.^8^ [^18^F]fluoromethylcholine, which is typically utilised in prostate cancer staging to identify choline uptake, may also be useful to identify concurrent AAA in patients with prostate cancer.^9^ Regarding the prospect of SPECT, RP805, a [^99m^Tc]‐labelled radiotracer that targets matrix metalloproteinases (MMPs), has been used to demonstrate that MMP‐targeted SPECT may indicate aortic wall inflammation and help predict expansion or rupture in AAA.^10^ Furthermore, a novel pan‐MMP tracer called RYM1 has successfully demonstrated the specific detection of MMPs and inflammatory activity in AAA.^11^ Ultimately, with clinical implementation, the findings of these radiotracers may perhaps replace the use of [^18^F]FDG uptake and supplement classical clinical parameters, such as aneurysm diameter, since AAA growth—and consequently rupture—is implied to follow a non‐linear and unpredictable trend. As PET and SPECT are currently used in the clinic for non‐AAA applications, further research on these molecular imaging modalities for patients with AAA has functional practicality for rapid clinical translation compared to the process of translating entirely novel technologies to the clinic.

**FIGURE 1:**
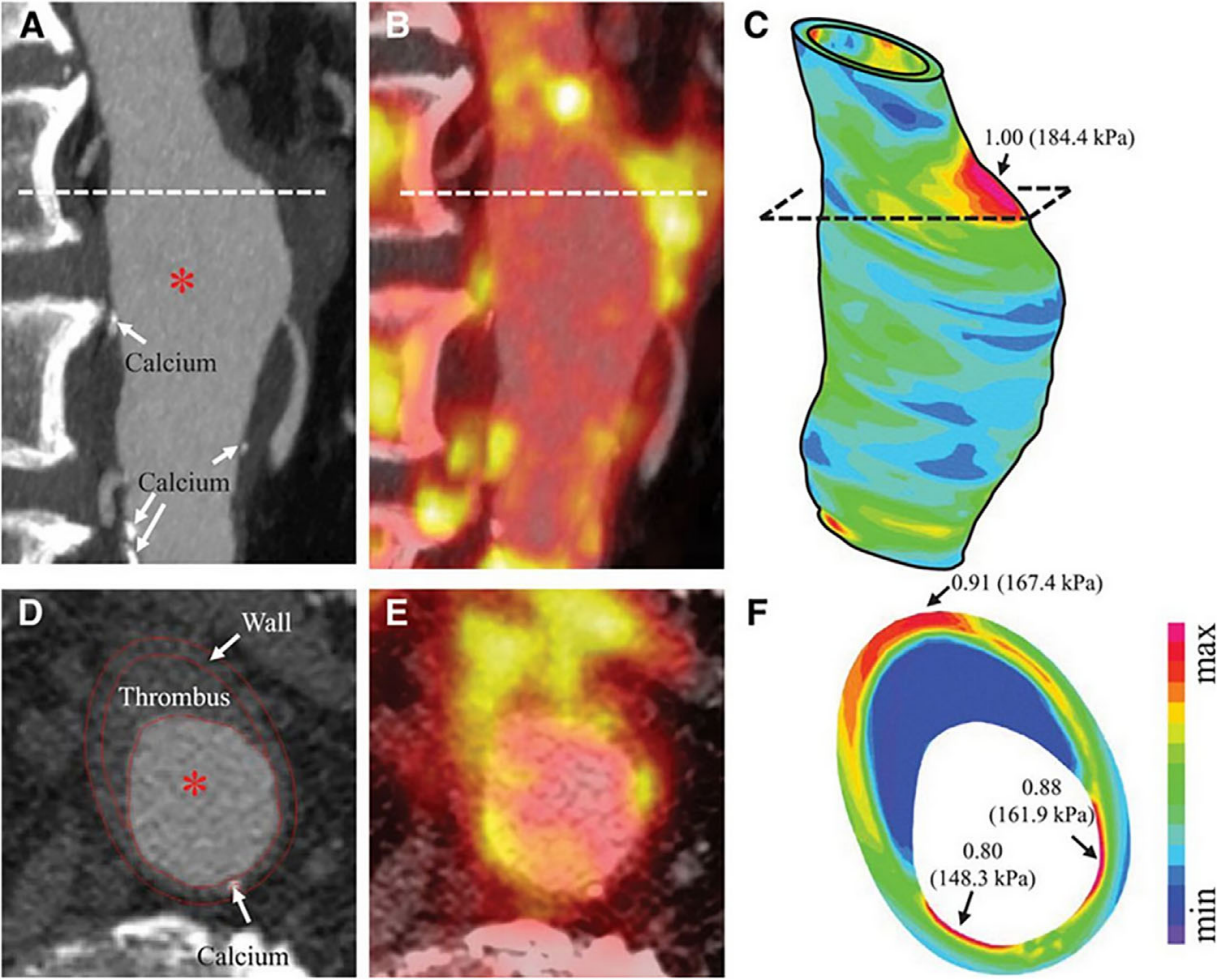
In vivo imaging and structural stress of abdominal aortic aneurysms (AAA). Sagittal‐view (A) contrast‐enhanced CT and (B) [^18^F]FDG PET, with (C) structural stress plotted on 3D geometry. Transverse‐view (D) contrast‐enhanced CT and (E) [^18^F]FDG PET, with (F) structural stress plotted on the transverse plane.^13^ Figure license: Huang et al., 2016. High structural stress andpresence of intraluminal thrombus predict abdominal aortic aneurysm 18F‐FDGuptake. This article is distributed under the terms of the Creative CommonsAttribution 4.0 International License (https://creativecommons.org/licenses/by/4.0/). Abbreviations: AAA, abdominal aortic aneurysm; CT, computed tomography; [^18^F]FDG, [^18^F]fluorodeoxyglucose; PET, positron emission tomography.

**FIGURE 2:**
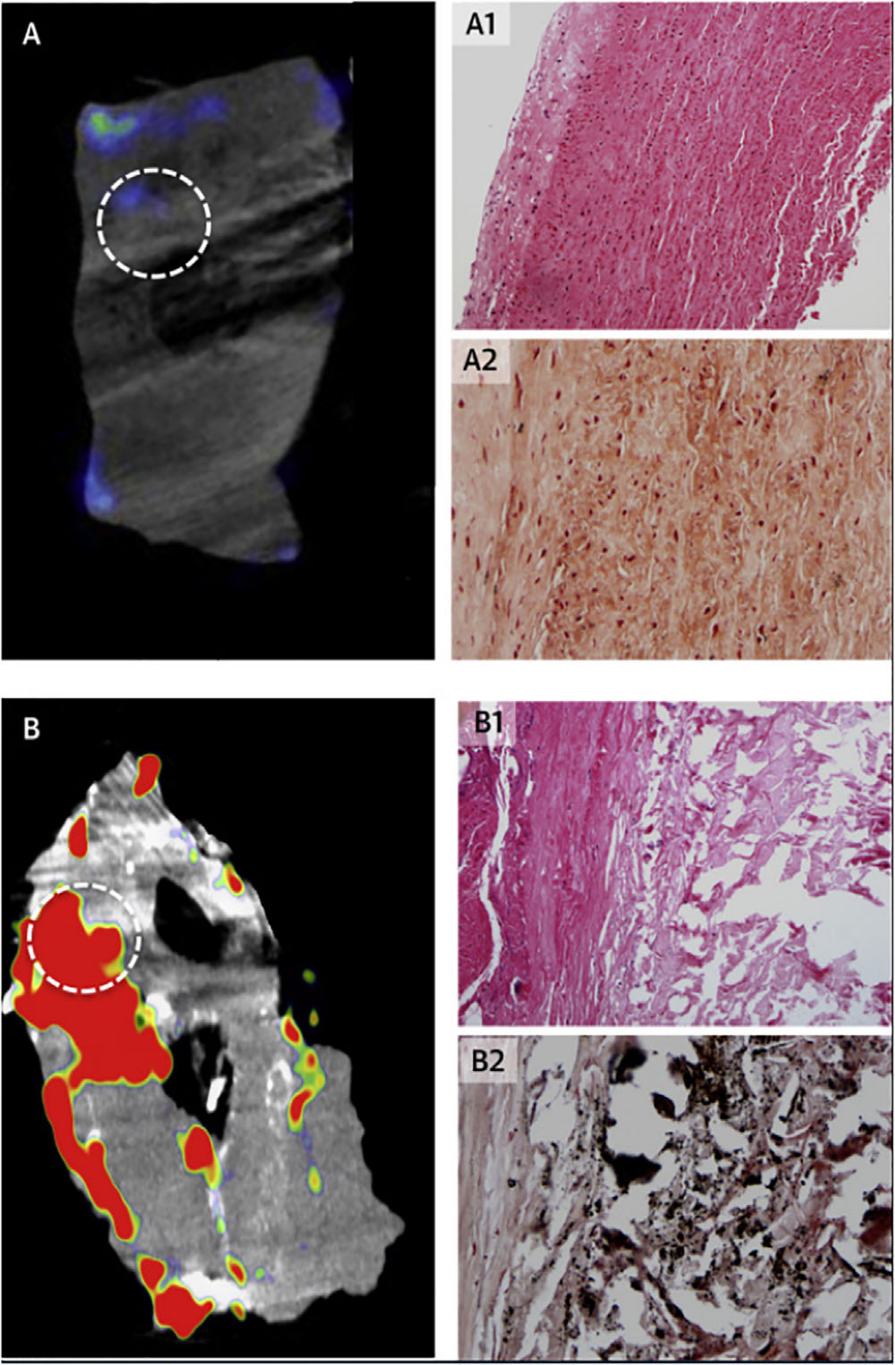
Ex vivo [^18^F]NaF micro‐PET/CT images and histology of aortic wall from (A) a patient without an aneurysm at post‐mortem and (B) a patient during open abdominal aortic aneurysm (AAA) repair. Regions of interest (dashed circle) of [^18^F]NaF uptake demonstrate (B1) atheromatous disease with necrosis (haematoxylin and eosin stain, magnification × 100) and (B2) calcification (black, Von Kossa stain, magnification × 200) in the AAA tissue that is not apparent in the control aorta (A1, A2).^6^ Figure license: Forsythe et al., 2018. 18 F‐sodiumfluoride uptake in abdominal aortic aneurysms: The SoFIA 3 study. Thisarticle is distributed under the terms of the Creative Commons Attribution 4.0International License (https://creativecommons.org/licenses/by/4.0/). Abbreviations: AAA, abdominal aortic aneurysm; [^18^F]NaF, [^18^F]‐sodium fluoride; PET/CT, positron emission tomography/computed tomography.

**FIGURE 3:**
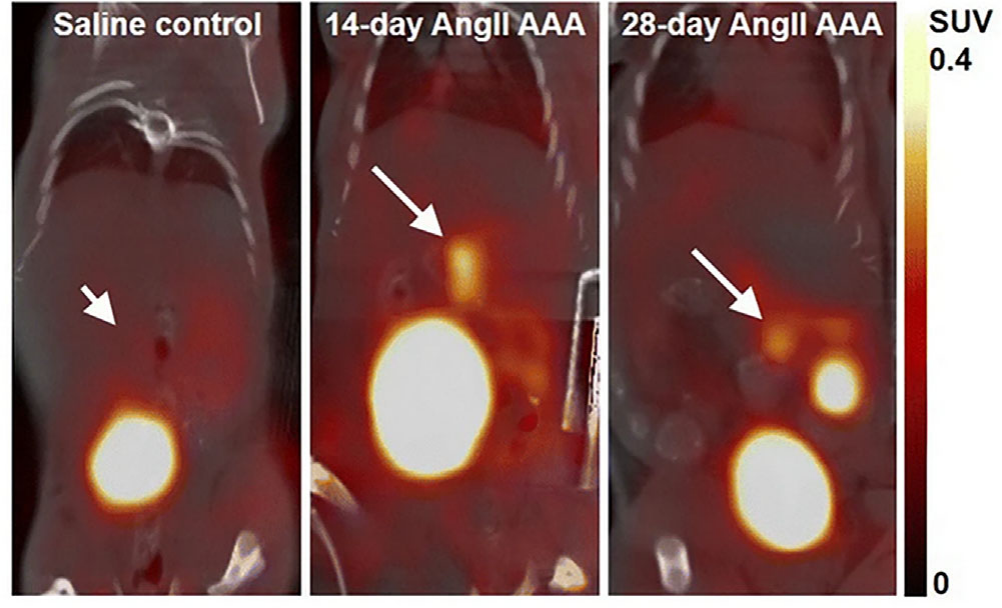
Representative coronal‐view static [^18^F]FLT PET/CT images. White arrows indicate abdominal aortic location (short arrow, saline control; long arrow, abdominal aortic aneurysm [AAA]).^7^ Figure license: Gandhi et al., 2019. Cell proliferation detectedusing [18F]FLT PET/CT as an early marker of abdominal aorticaneurysm. This article is distributed under the terms of the Creative CommonsAttribution 4.0 International License (https://creativecommons.org/licenses/by/4.0/). Abbreviations: [^18^F]FLT, [^18^F]fluorothymidine; PET/CT, positron emission tomography/computed tomography.

The road ahead for molecular imaging of AAA may be intricate, and the smooth translation of positive preclinical findings to the clinic remains a critical aim. The key obstacle to implementing SPECT for AAA imaging in the clinic lies in the relatively poorer spatial resolution of clinical SPECT scanners than that of clinical PET scanners. However, if a SPECT radiotracer provides substantially valuable information, a dedicated redesign of collimators for AAA may enable higher‐resolution clinical SPECT. Meanwhile, in addition to the extensive range of PET radiotracers already available in the market, the development of PET scanners with a substantially longer axial field of view (4–8 times longer than that of the currently most commonly available PET scanners) offers a unique opportunity in accelerating the translation of new radiotracers in the clinic.^12^ Particularly, the unprecedentedly high image quality and resolution combined with the potential for substantial reduction in the injected radioactive dose to patients with suspected AAA will enable dynamic imaging as a powerful and sensitive tool to obtain quantitative and reproducible kinetic information that may help identify the disease at earlier stages and increase the efficacy of non‐invasive therapeutic interventions for patients with AAA.

In conclusion, many aspects of AAA evolution remain poorly understood. Considering the seemingly complex heterogeneity of AAA and involvement of numerous contributory factors, anatomical and molecular imaging modalities must be considered supplementary tools in their role in AAA management. Importantly, the strength of radionuclide‐based molecular imaging is reflected in its power to shed light on the molecular evolution of AAA, which may guide the necessity for surgical repair or response to pharmacological therapies before the disease progresses to a severe and irreversible stage. To facilitate AAA risk stratification, the quest to identify the most appropriate radiotracers is an essential path towards reducing patient morbidity and identifying alternative therapies that are potentially more efficient and economical than surgical interventions.

## FUNDING INFORMATION

Richa Gandhi is funded by the Engineering and Physical Sciences Research Council. Marc Bailey is funded by the British Heart Foundation. Charalampos Tsoumpas is funded by the Royal Society.

## CONFLICT OF INTEREST

The authors declare that there is no conflict of interest that could be perceived as prejudicing the impartiality of the research reported.
